# Dual BLyS/APRIL targeted therapy with telitacicept in rituximab-refractory SLE-associated neuromyelitis optica spectrum disorder: a case report

**DOI:** 10.3389/fimmu.2025.1602800

**Published:** 2025-05-26

**Authors:** Mingfen Dai, Chunyan Huang, Mingxuan Zhou, Pui-Ying Leong, Xiaoqing Chen

**Affiliations:** ^1^ Department of General Practice, The Second Affiliated Hospital of Fujian Medical University, Quanzhou, Fujian, China; ^2^ Department of Rheumatology and Immunology, The Second Affiliated Hospital of Fujian Medical University, Quanzhou, Fujian, China; ^3^ Institute of Medicine, Chung Shan Medical University, Taichung, Taiwan; ^4^ Division of Allergy, Immunology & Rheumatology, Department of Internal Medicine, Chung Shan Medical University Hospital, Taichung, Taiwan; ^5^ School of Medicine, Chung Shan Medical University, Taichung, Taiwan; ^6^ PhD Program of Business, Feng Chia University, Taichung, Taiwan

**Keywords:** neuromyelitis optica spectrum disorder, systemic lupus erythematosus, area postrema syndrome, optic neuritis, AQP4-IgG, telitacicept

## Abstract

This article presents a case of neuromyelitis optica spectrum disorder (NMOSD) secondary to systemic lupus erythematosus (SLE). The patient initially presented with unexplained nausea, vomiting, intractable hiccups, and significant bradycardia (48 bpm). Cranial and spinal MRI findings were unremarkable, while serum aquaporin-4 immunoglobulin G (AQP4-IgG) antibody titers were markedly elevated (1:320). Following exclusion of alternative etiologies through comprehensive serological and neuroimaging investigations, the diagnosis of area postrema syndrome (APS) was confirmed according to the 2015 International Panel for NMO Diagnosis (IPND) criteria as a distinct NMOSD subtype. The patient exhibited a rapid therapeutic response to high-dose glucocorticoid therapy and was discharged on maintenance methylprednisolone with adjunctive medications. At one-month follow-up, the patient reported progressive visual deterioration, prompting neuro-ophthalmologic evaluation. Clinical findings included mildly elevated intraocular pressure (22.2 mmHg OD, 22.5 mmHg OS), corrected refractive error, and abnormal visual evoked potentials (diminished amplitude with delayed waveforms). Other neuro-ophthalmic investigations revealed no abnormalities. Persistent AQP4-IgG seropositivity (titer 1:32) was noted, and gadolinium-enhanced MRI revealed focal signal abnormalities in the bilateral optic nerves, confirming optic neuritis. Intravenous rituximab therapy (500 mg every two weeks) was initiated; however, treatment was complicated by a generalized urticarial rash and pleuritic chest pain, with no significant improvement in visual acuity. The therapeutic regimen was subsequently modified to incorporate subcutaneous telitacicept (160 mg weekly) in combination with glucocorticoid taper protocol and hydroxychloroquine. After eight weeks of this combined therapy, marked visual improvement was observed. Follow-up gadolinium-enhanced MRI revealed decreased enhancement intensity in the corresponding optic nerve regions compared to baseline. The patient maintained clinical stability and continues long-term multidisciplinary surveillance. To our knowledge, this represents the third documented case validating the therapeutic efficacy of telitacicept in NMOSD. Our findings suggest that telitacicept may serve as a disease-modifying therapy for SLE patients with AQP4-IgG-seropositive NMOSD.

## Introduction

Neuromyelitis optica spectrum disorder (NMOSD) is a rare autoimmune demyelinating disease characterized by multifocal inflammatory lesions in the central nervous system (CNS), predominantly targeting aquaporin-4-rich regions (1). The 2015 International Panel for NMO Diagnosis (IPND) defines six core clinical phenotypes, with longitudinally extensive transverse myelitis (LETM), area postrema syndrome (APS), and optic neuritis representing the most prevalent manifestations. APS typically presents with refractory nausea/vomiting and intractable hiccups, often preceding other neurological deficits. Multifocal CNS involvement may precipitate synchronous or rapidly-sequential clinical syndromes, most characteristically the concurrence of optic neuritis and myelitis (1).

Pathogenetically, NMOSD is driven by serum aquaporin-4 immunoglobulin G (AQP4-IgG) antibodies, detectable in about 80% of cases (2). Approximately 30-40% of AQP4-IgG seropositive patients exhibit concurrent autoimmune disorders, with systemic lupus erythematosus (SLE) being the most prevalent comorbidity (1). The coexistence of SLE and NMOSD imposes substantial diagnostic and therapeutic challenges, compounded by the paucity of evidence-based management guidelines. Current strategies predominantly derive from NMOSD clinical trials, underscoring the imperative for targeted therapies addressing both conditions (3). Here, we present a case of SLE-associated NMOSD where telitacicept therapy achieved sustained remission following rituximab-refractory optic neuritis.

## Case presentation

A 33-year-old woman fulfilling the 2019 EULAR/ACR classification criteria for SLE presented in August 2019 with thrombocytopenia (platelet count 4×10^9^/L), hypocomplementemia (C3 0.60 g/L, C4 0.13 g/L), antinuclear antibody positivity (ANA titer 1:100, speckled pattern), and elevated anti-β2-glycoprotein I antibodies (40.77 RU/mL). Induction therapy with methylprednisolone (500 mg/day IV for 3 days), intravenous immunoglobulin (IVIg; 0.4 g/kg/day), and hydroxychloroquine (HCQ; 200 mg bid) normalized platelet counts. Maintenance prednisone (5–10 mg alternate days) and HCQ (200mg qd) was self-discontinued during lactation in March 2024.

In April 2024, the patient presented with 48-hour persistent nausea/vomiting. Physical examinations were unremarkable. Laboratory investigations revealed severe thrombocytopenia (platelet count 13×10^9^/L) and hypokalemia (serum potassium 2.9 mmol/L). ANA serology showed a positive titer of 1:100 with a speckled pattern, and anti-SSA (+++) and anti-RO52 (+++) antibodies were detected, while anti-dsDNA antibody was negative. Additional assessments, including urinalysis, stool analysis, C-reactive protein (CRP), erythrocyte sedimentation rate (ESR), lupus anticoagulant, antiphospholipid syndrome markers, direct antiglobulin test, and complement C3/C4 levels, were within normal limits. Imaging studies (head, chest, and abdominopelvic CT scans) revealed no pathological findings. Based on the patient’s clinical manifestations, the SLE Disease Activity Index 2000 (SLEDAI-2K) score was 1 (4). Combination therapy with methylprednisolone (60 mg qd), cyclosporine (75 mg bid), HCQ (200 mg qd), IVIg (0.4 g/kg/day IV for 3 days), and rabeprazole (20 mg qd) increased platelets to 93×10^9^/L within 5 days.

However, the patient persisted with refractory symptoms including severe nausea, vomiting (rendering oral intake impossible), intractable hiccups, dizziness, chest tightness, and abrupt-onset sinus bradycardia (48 bpm; baseline 70–80 bpm), without diarrhea, melena, or neurological deficits. Physical examination revealed no localized abdominal tenderness or peritoneal signs, no cardiac murmurs or signs of heart failure on cardiovascular assessment, and demonstrated intact cranial nerves, normal motor strength, and absence of sensory deficits on neurological evaluation. Electrocardiogram confirmed sustained sinus bradycardia, while cardiac biomarkers (troponin T, N-terminal pro-brain natriuretic peptide, myocardial enzymes) and echocardiography showed no abnormalities. Repeat abdominal imaging (CT and ultrasound) ruled out acute pathology, and upper endoscopy identified minor mucosal injuries: a linear tear at the lesser curvature of the cardia and a superficial gastric ulcer. Despite aggressive supportive care—including fasting, nasogastric decompression, gastroprotective agents (high-dose proton pump inhibitors), antiemetics (metoclopramide and ondansetron), and fluid resuscitation—symptoms persisted without improvement.

Given the refractory nature of symptoms, neurological etiologies were investigated. Brain and cervicothoracic spine MRI revealed only mild C4/5 disc protrusion without demyelinating lesions, while serological testing demonstrated strongly positive AQP4-IgG antibodies (titer 1:320 via cell-based assay). In accordance with the IPND (2015) criteria (3), a diagnosis of NMOSD with area postrema syndrome was established based on the presence of core clinical features (APS), high-titer AQP4-IgG seropositivity, and exclusion of alternative diagnoses (neoplastic, metabolic, or infectious causes).

The patient received intravenous methylprednisolone pulse therapy (500 mg/day for 3 days), adjunctive baclofen for diaphragmatic spasm relief, and intensified gastroprotection, resulting in rapid symptom resolution. Following discharge, maintenance immunosuppression with oral methylprednisolone (tapered regimen), HCQ, and cyclosporine was initiated under close multidisciplinary monitoring.

**Figure 1:**
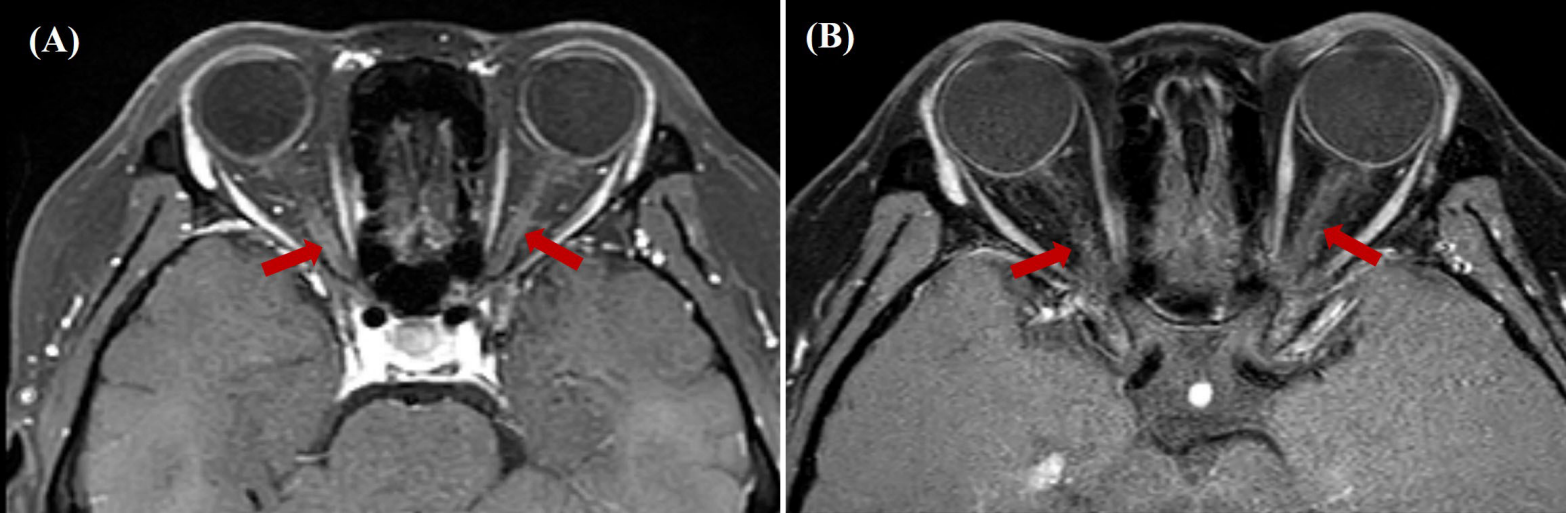
**(A)** Pre-treatment gadolinium-enhanced orbital MRI (axial STIR sequence) revealed bilateral optic nerve thickening and patchy, heterogeneous enhancement. **(B)** Post-treatment gadolinium-enhanced orbital MRI (axial STIR sequence) revealed decreased enhancement intensity in the corresponding optic nerve regions compared to baseline. STIR, Short Tau Inversion Recovery.

During follow-up in June 2024, the patient reported clinically significant visual impairment exacerbated by near work. Retrospective history revealed previously undocumented subclinical visual symptoms during the acute APS episode. Comprehensive ophthalmological assessment demonstrated mildly elevated intraocular pressure (22.2 mmHg OD, 22.5 mmHg OS), uncorrected refractive error, and delayed visual evoked potentials with amplitude reduction (diminished amplitude with delayed waveforms). Orbital MRI revealed focal inflammatory changes bilaterally in the optic nerves (**Figure 1**), confirming optic neuritis. Subsequent serological testing revealed an AQP4-IgG antibody titer of 1:32 with elevated levels of CD19+ B cells (30.07%; 757.78/μL). Immunotherapy was initiated with rituximab (500 mg IV q2w). The initial infusion resulted in moderate visual acuity improvement, however, the subsequent administration failed to demonstrate clinical efficacy and was complicated by cutaneous hypersensitivity reactions accompanied by chest discomfort. Therapeutic strategy was therefore modified to telitacicept (160 mg administered subcutaneously weekly) in combination with glucocorticoids and HCQ.

**Figure 2:**
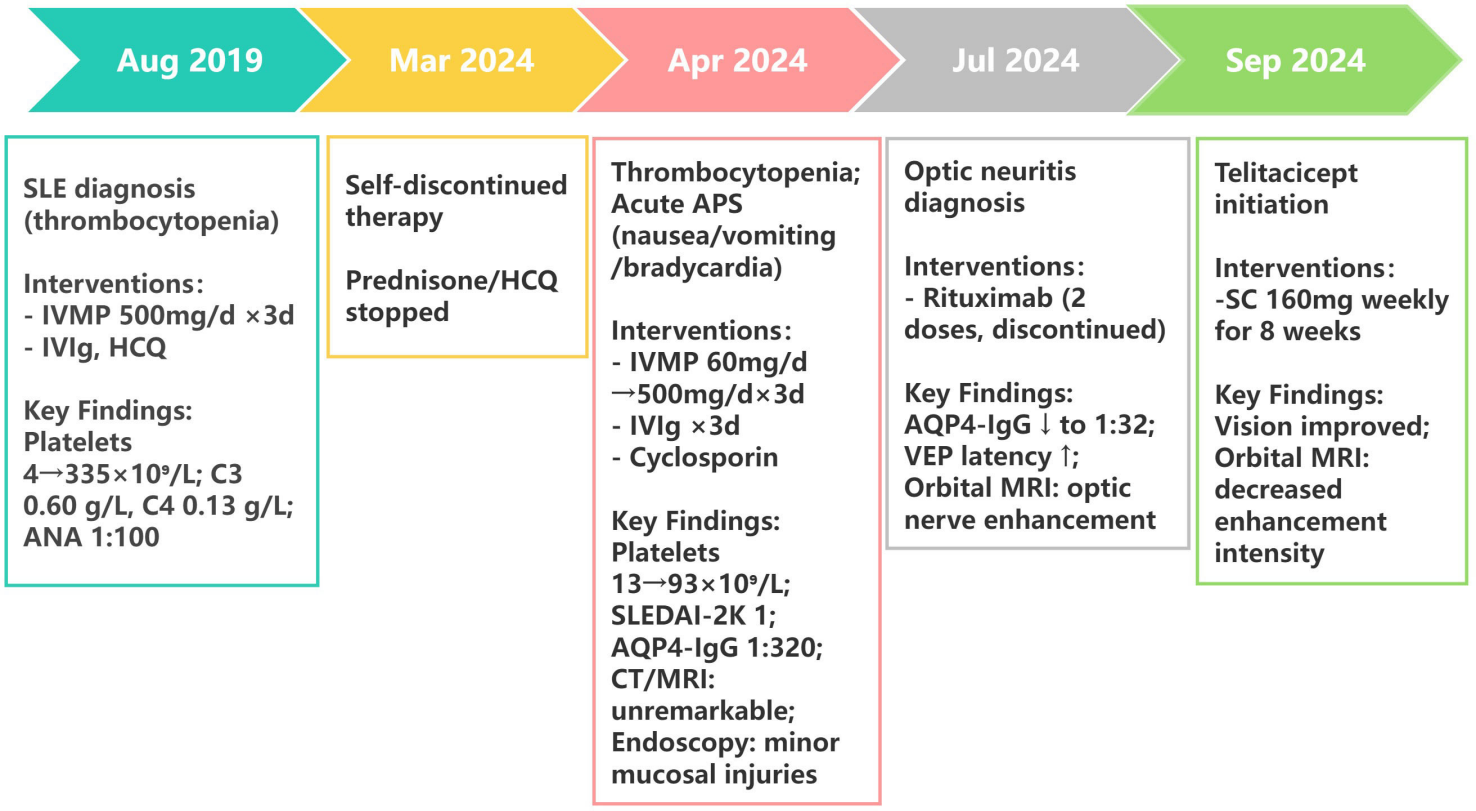
Clinical timeline of SLE-NMOSD overlap case with treatment response. SLEDAI-2K, Systemic Lupus Erythematosus Disease Activity Index 2000; IVMP, intravenous methylprednisolone; IVIg, intravenous immunoglobulin; HCQ, hydroxychloroquine; VEP, visual evoked potential.

Following 8-week telitacicept therapy, the patient reported marked improvement in visual function. No injection-site reactions were reported during subcutaneous telitacicept administration. Post-treatment MRI demonstrated decreased gadolinium enhancement of optic nerves compared to pre-treatment baseline imaging (**Figure 1B**). The disease trajectory is summarized in **Figure 2**, illustrating the progression from SLE onset to NMOSD complications and treatment responses. The patient’s clinical status remains stable under continuous clinical surveillance.

## Discussion

Current evidence supports a well-established association between NMOSD and concurrent autoimmune diseases, including SLE. However, due to the timing of the development of classification criteria, the 1999 ACR neuropsychiatric lupus (NPSLE) criteria (5) did not formally include NMOSD, contributing to underdiagnosis in SLE cohorts and therapeutic challenges. Patients with such comorbidities typically manifest classic NMOSD phenotypes (APS, optic neuritis, or LETM) (6), but distinguishing SLE-related CNS involvement from coexisting NMOSD remains challenging due to overlapping features and the lack of standardized attribution criteria. Anna et al. (7) previously differentiated SLE myelitis from SLE-overlapping NMOSD. They observed that SLE myelitis typically occurs early in the disease course (within 1–2 years of SLE onset), whereas SLE-overlapping NMOSD tends to be late-onset and independent of SLE disease activity. Laboratory markers such as anti-dsDNA antibodies, complement levels, and antiphospholipid antibodies may remain normal in such cases, a pattern consistent with that observed in our patient. The presence of AQP4-IgG seropositivity and LETM may help differentiate NMOSD from SLE-associated myelitis (7). Future revisions of the NPSLE classification criteria should consider incorporating NMOSD.

Notably, clinical observations indicate that isolated APS, which is frequently misdiagnosed as gastroenterological disorders (8), precedes spinal/optic nerve inflammation in 58%–68% of NMOSD cases (9). This temporal relationship highlights the need for early serological testing (AQP4-IgG) in SLE patients with treatment-refractory symptoms. In this case, an accurate diagnosis was achieved during the early disease phase despite initial oversight of subtle visual disturbances (blurred vision) unreported by the patient. The prompt therapeutic response following aggressive immunosuppression underscores the critical importance of considering NMOSD in SLE patients presenting with intractable gastrointestinal symptoms.

Of particular interest is the patient’s concomitant symptomatic sinus bradycardia (48 bpm). Emerging case reports have documented NMOSD-associated cardiac manifestations, including sick sinus syndrome (SSS) (10, 11). This observation suggests that the triad of refractory nausea/vomiting, abrupt-onset bradycardia, and autonomic dysfunction may serve as diagnostic red flags for NMOSD, necessitating heightened clinical vigilance.

Current evidence regarding the association between NMOSD and SLE remains limited to case series and cohort studies, with therapeutic protocols predominantly derived from NMOSD clinical trials (3). Acute management typically involves pulse methylprednisolone (1 g/day IV for 5 days) combined with adjunctive therapies including plasma exchange, IVIg, and immunoadsorption. Current guidelines advocate early initiation of immunosuppressive regimens (e.g., mycophenolate mofetil and azathioprine) combined with B-cell depletion therapy—specifically rituximab—to mitigate relapse rates. Given the established pathophysiological correlation between B lymphocyte-derived anti-AQP4 antibodies and disease relapse in NMOSD, Shidahara et al. (12) documented successful rituximab administration in AQP4-IgG-positive SLE-NMOSD overlap syndrome, highlighting its therapeutic potential in refractory cases.

Our patient manifested inadequate therapeutic response to two rituximab infusions with concurrent development of grade 2 cutaneous hypersensitivity reactions. Therapeutic strategy was consequently modified to telitacicept, a novel recombinant fusion protein targeting both B lymphocyte stimulator (BLyS) and a proliferation-inducing ligand (APRIL) (13). This dual mechanism suppresses B-cell maturation and T-cell co-stimulation, offering potential therapeutic synergy for addressing the pathogenesis of SLE and NMOSD.

Telitacicept received conditional approval from China’s National Medical Products Administration (NMPA) in March 2021 for the treatment of SLE (Approval No. S20210008) (13). The phase IIb randomized controlled trial (NCT02885610) (14) provided robust evidence supporting its efficacy and safety profile. Based on positive phase III clinical trial outcomes, the NMPA granted full marketing authorization in November 2023, marking its transition from conditional to full approval status. Despite its established role in SLE, literature on telitacicept’s application in NMOSD remains limited. In 2023, Jie Ding et al. (15) conducted the first exploratory study involving eight relapsing NMOSD patients who underwent plasmapheresis followed by 46 weeks of weekly subcutaneous telitacicept (240 mg). Preliminary results indicated prolonged relapse-free intervals and reduced annualized relapse rates (ARR), though the small sample size necessitates validation through larger trials. Further supporting its potential, Li et al. (16) reported sustained remission in a complex NMOSD case with comorbid Sjögren’s syndrome and autoimmune hepatitis–primary biliary cholangitis after 14 months of telitacicept maintenance therapy. Notably, an ongoing phase III trial (NCT03330418) is evaluating telitacicept versus placebo in 118 NMOSD patients (13), underscoring the growing interest in its therapeutic utility.

Although not currently included in NMOSD treatment guidelines, telitacicept (160 mg SC weekly) was added to the patient’s baseline immunosuppressive regimen following ethical committee approval and informed consent, based on its established efficacy in SLE and emerging NMOSD evidence. Close monitoring revealed no significant adverse events, and post-8-week evaluations showed improved best-corrected visual acuity alongside reduced optic nerve gadolinium enhancement on MRI (**Figure 1**). While rituximab was initiated to target CD20^+^ B cells, its discontinuation due to hypersensitivity precluded sustained depletion. The subsequent clinical improvement (resolved optic neuritis) coincided with telitacicept initiation, which inhibits BAFF/APRIL to suppress plasma cell survival and autoantibody production. Given the rapid timeline (8 weeks) and lack of post-rituximab B-cell monitoring, telitacicept’s mechanism—rather than residual rituximab effects—likely drove the remission. This clinical trajectory positions telitacicept as a promising alternative for rituximab-refractory SLE-NMOSD overlap syndrome, bridging mechanistic innovation with practical therapeutic gains.

Long-term maintenance therapy with telitacicept will be continued, with quarterly monitoring of CD19+ lymphocytes and serum IgG levels. Multicenter prospective studies are imperative to establish evidence-based guidelines for biologics in autoimmune overlap syndromes.

## Data Availability

The original contributions presented in the study are included in the article/supplementary material. Further inquiries can be directed to the corresponding authors.

## References

[1] Wingerchuk DM Lucchinetti CF. Neuromyelitis optica spectrum disorder. N Engl J Med. (2022) 387:631–9. doi: 10.1056/NEJMra1904655 36070711

[2] Schindler P Aktas O Ringelstein M Wildemann B Jarius S Paul F. Glial fibrillary acidic protein as a biomarker in neuromyelitis optica spectrum disorder: a current review. Expert Rev Clin Immunol. (2023) 19:71–91. doi: 10.1080/1744666X.2023.2148657 36378751

[3] Kopp CR Prasad CB Naidu S Sharman V Misra DP Agarwal V. Overlap syndrome of anti-aquaporin-4 positive neuromyelitis optica spectrum disorder and systemic lupus erythematosus: A systematic review of individual patient data. Lupus. (2023) 32:1164–72. doi: 10.1177/09612033231191180 37487596

[4] Chitpet P Chaiamnuay S Narongroeknawin P Asavatanabodee P Leosuthamas P Pakchotanon R. The effect of systemic lupus erythematosus (SLE) Disease Activity Score and SLE Disease Activity Index 2000-based remission states in patients with SLE on damage accrual. Int J Rheum Dis. (2023) 26:2509–16. doi: 10.1111/1756-185X.14949 37875327

[5] Liang MH Corzillius M Bae SC Lew RA Fortin PR Gordon C. The American College of Rheumatology nomenclature and case definitions for neuropsychiatric lupus syndromes. Arthritis Rheumatol. (1999) 42:599–608. doi: 10.1002/1529-0131(199904)42:4<599::AID-ANR2>3.0.CO;2-F 10211873

[6] Martín-Nares E Hernandez-Molina G Fragoso-Loyo H. Aquaporin-4-IgG positive neuromyelitis optica spectrum disorder and systemic autoimmune diseases overlap syndrome: a single-center experience. Lupus. (2019) 28:1302–11. doi: 10.1177/0961203319877255 31566079

[7] Abou RA Raya SA. Neuromyelitis optica spectrum disorders (NMOSD) and systemic lupus erythematosus (SLE): Dangerous duo. Int J Rheum Dis. (2024) 27:e14973. doi: 10.1111/1756-185X.14973 37975635

[8] Liu T Li L Guo X Li Q Jia D Ma L. Clinical analysis of neuromyelitis optica spectrum disease with area postrema syndrome as the initial symptom. Eur J Med Res. (2022) 27:315. doi: 10.1186/s40001-022-00949-9 36582004 PMC9798654

[9] Siriratnam P Huda S Butzkueven H van der Walt A Jokubaitis V Monif M. A comprehensive review of the advances in neuromyelitis optica spectrum disorder. Autoimmun Rev. (2023) 22:103465. doi: 10.1016/j.autrev.2023.103465 37852514

[10] Hamaguchi M Fujita H Suzuki T Suzuki K. Sick sinus syndrome as the initial manifestation of neuromyelitis optica spectrum disorder: a case report. BMC Neurol. (2022) 22:56. doi: 10.1186/s12883-022-02580-x 35164681 PMC8842888

[11] Lin H Duan X Li L Ye J Xiao H. Neuromyelitis optica spectrum disorder with sick sinus syndrome: two cases and a literature review. Healthc (Basel). (2023) 11:2810. doi: 10.3390/healthcare11212810 PMC10649943 37957955

[12] Shidahara K Hayashi K Sada KE Hiramatsu S Morishita M Watanabe H. Refractory neuromyelitis optica spectrum disorder in systemic lupus erythematosus successfully treated with rituximab. Lupus. (2018) 27:1374–7. doi: 10.1177/0961203318760994 29498304

[13] Dhillon S. Telitacicept: first approval. Drugs. (2021) 81:1671–5. doi: 10.1007/s40265-021-01591-1 34463932

[14] Wu D Li J Xu D Merrill JT van Vollenhoven RF Liu Y. Huang C et al: Telitacicept in patients with active systemic lupus erythematosus: results of a phase 2b, randomised, double-blind, placebo-controlled trial. Ann Rheum Dis. (2024) 83:475–87. doi: 10.1136/ard-2023-224854 PMC10958275 38129117

[15] Ding J Jiang X Cai Y Pan S Deng Y Gao M. Telitacicept following plasma exchange in the treatment of subjects with recurrent neuromyelitis optica spectrum disorders: A single-center, single-arm, open-label study. CNS Neurosci Ther. (2022) 28:1613–23. doi: 10.1111/cns.13904 PMC9437241 35851754

[16] Li F Sui X Pan X Liu C Xie L Zhao H. Neuromyelitis optica spectrum disorder with ultra-longitudinally extensive transverse myelitis: A case report and literature review. Heliyon. (2024) 10:e39687. doi: 10.1016/j.heliyon.2024.e39687 39559196 PMC11570508

